# Clinical relevance of pulmonary non-tuberculous mycobacterial isolates in three reference centres in Belgium: a multicentre retrospective analysis

**DOI:** 10.1186/s12879-019-4683-y

**Published:** 2019-12-17

**Authors:** Yannick Vande Weygaerde, Nina Cardinaels, Peter Bomans, Taeyang Chin, Jerina Boelens, Emmanuel André, Eva Van Braeckel, Natalie Lorent

**Affiliations:** 1 Department of Respiratory Medicine, Ghent University Hospital, Corneel Heymanslaan 10, B9000 Ghent, Belgium; 2 Department of Respiratory Diseases, University Hospitals Leuven, Herestraat 49, B3000 Leuven, Belgium; 3 Department of Pneumology, Antwerp Hospital Network Stuivenberg, Lange Beeldekensstraat 267, B2060 Antwerp, Belgium; 4 Department of Laboratory Medicine, Department of Clinical Chemistry, Microbiology and Immunology, Ghent University Hospital, Corneel Heymanslaan 10, B9000 Ghent, Belgium; 5 Laboratory of Clinical Bacteriology and Mycology, Department of Microbiology, Immunology and Transplantation, University Hospitals Leuven, Herestraat 49, Leuven, B3000 Belgium

**Keywords:** Non-tuberculous mycobacteria (NTM), Non-tuberculous mycobacterial pulmonary disease (NTM-PD), Epidemiology, Radiology

## Abstract

**Background/objectives:**

Assessing the clinical relevance of non-tuberculous mycobacteria (NTM) isolated from respiratory samples can be challenging. The epidemiology and pathogenicity of NTM species vary geographically. We aimed to outline the clinical relevance and associated radiological patterns of NTM species isolated in Belgium.

**Methods:**

We performed a retrospective multicentre analysis of all patients identified from the laboratory database with > 1 respiratory sample growing NTM from January 2010 through December 2017. We collected clinical, radiological and microbiological data through medical record review and assessed clinical relevance according to ATS/IDSA criteria for NTM pulmonary disease (NTM-PD).

**Results:**

Of the 384 unique patients, 60% were male, 56% had a smoking history and 61% had pre-existing lung disease. *Mycobacterium avium* complex (MAC), *M. gordonae* and *M. xenopi* were the most frequently isolated species: 53, 15 and 8% respectively. 43% of patients met ATS/IDSA criteria, of whom 28% presented with fibrocavitary disease. Weight loss, fever, nodular bronchiectatic and fibrocavitary lesions on chest CT, and a positive acid-fast bacilli (AFB) stain were significantly associated with NTM-PD. The species with the highest pathogenic potential were *M. abscessus* (11/12), *M. malmoense* (6/7) and *M. intracellulare* (41/64).

**Conclusion:**

In our study, MAC was the most commonly isolated NTM species, but *M. abscessus* and *M. malmoense* showed the highest probability of being clinically relevant. Clinical relevance varied not only by species but also by radiological findings on chest CT and AFB staining. Clinicians should consider these elements in their treatment decision making. Prospective data including clinical outcome are needed to provide more robust evidence.

## Background

Non-tuberculous mycobacteria (NTM) are ubiquitous in the natural environment and household water systems [1, 2]. Over 180 different species have been identified, but only 32 have been reported causing human or animal disease [3]. Lung infections (NTM related pulmonary disease or NTM-PD) account for 90% of NTM related disease. Patients with pre-existing lung disease like chronic obstructive pulmonary disease (COPD), cystic fibrosis (CF), and non-CF bronchiectasis are predisposed [4–8]. To a lesser extent NTM can cause infections of skin, sinuses, lymph nodes, or even lead to disseminated disease in case of innate or acquired immunodeficiency [5, 9].

Worldwide pulmonary isolation of NTM is increasing and, according to some data, so is the incidence of NTM-PD [10–14]. Since NTM-PD is not a reportable disease in most countries, reliable epidemiological data are scarce.

NTM species vary between countries and regions and can even change over time, as does their respective pathogenicity [10, 12, 15–17]. Overall, species from the *Mycobacterium avium* complex (MAC) are the most commonly isolated NTM [12, 15, 18]. *M. avium* predominates in North America [12, 13], whereas in Australia *M. intracellulare* is more prevalent [19]. In the Republic of Korea *M. intracellulare* is the most prevalent species but *M. abscessus* is also quite common [20, 21]. In Europe, *M. avium* and *M. intracellulare* are almost equally prevalent, but species other than MAC are also frequently isolated which can be clinically relevant as well (*M. kansasii*, *M. xenopi, M. malmoense, ...)* [15, 18]. There are marked regional differences in isolation frequency and pathogenic potential of different species[12, 15, 18]. Epidemiological data from Belgium are limited to isolation frequency without clinical information [17].

Assessing the clinical relevance of respiratory NTM isolates can be challenging. Intermittent isolation is frequent and spontaneous culture conversion is possible without harm to the patient. The American Thoracic Society (ATS) and the Infectious Diseases Society of America (IDSA) developed diagnostic criteria for NTM-PD to guide treatment decisions, since objective markers of disease predicting which patients will develop active NTM-PD are lacking [4]. These criteria are widely implemented although based on expert opinion and barely validated [5]. Whether they can be universally applied in all patient populations remains debatable.

Besides ATS/IDSA criteria, taking into account the local epidemiology and individual species pathogenicity can facilitate treatment decision-making. This should be a well-considered process given the prolonged, potentially toxic and costly multidrug regimens used in NTM-PD with relatively poor efficacy [4, 5, 7].

We aimed to outline the epidemiology of respiratory NTM isolates in our region, assess their clinical relevance according to the ATS/IDSA criteria and explore the possibility of an association between clinical relevance and the radiological pattern on chest CT.

## Methods

### Design

We performed a multicentre, retrospective cohort study including all patients with at least one respiratory sample yielding NTM between January 2010 and December 2017 from three Belgian reference centres: University Hospitals Leuven, Ghent University Hospital and Stuivenberg Hospital Antwerp. The ethics committees of all participating centres granted approval. Informed consent was waived given the retrospective nature of the study.

### Study cohort

Starting from the microbiology database, we searched for samples positive for NTM of patients aged > 18 years from January 2010 through December 2017. We only included patients with respiratory samples defined as sputum, bronchial wash, mediastinal lymph node biopsy and lung biopsy. People living with human immunodeficiency virus (HIV) were excluded because of their different disease presentation.

### Data collection

At least one investigator per centre reviewed the electronic patient record using a standardised clinical report form. We collected the following data: age, gender, race, comorbidities, smoking history, clinical presentation, radiological features at diagnosis, microbiology (NTM species, acid-fast bacilli (AFB) staining, co-isolation of *Aspergillus fumigatus* species complex and/or *Pseudomonas aeruginosa*). Clinical relevance was defined in accordance with ATS/IDSA criteria for NTM-PD i.e. presence of symptoms and radiologic abnormalities compatible with NTM lung disease and microbiological confirmation as specified in Table 1. Cases who did not meet the ATS/IDSA diagnostic criteria were only considered as respiratory isolation of an NTM without NTM-PD. For challenging cases a consensus was sought through an expert panel of 3 senior investigators. Immunodeficiency was considered a comorbidity when the patient suffered from a primary immunodeficiency or haematological malignancy, when he was treated with immunosuppressive drugs (systemic corticosteroids, tumor necrosis alpha inhibitors, cytostatic agents) or had a solid organ transplant or bone marrow transplant [9]. Radiological features were defined as ‘fibrocavitary’ when fibrotic and/or cavitary lesions were present and ‘nodular bronchiectatic’ when multiple small nodules <5mm and/or bronchiectasis were found. All other anomalies were grouped as ‘other’. Respiratory samples were incubated on liquid and/or solid mycobacterial culture media. After ruling out the presence of *M. tuberculosis* complex (MTBc) using a targeted PCR amplifying the MTBc-specific IS6110, species identification was performed at the national reference laboratory for mycobacteriology (Sciensano, Brussels, Belgium) from positive cultures and was based on a combination of techniques targeting the 16S rRNA gene. PCR-based techniques included Inno-Lipa Mycobacteria v2 (Innogenetics, Gent, Belgium) and the Genotype Mycobacterium CM (Hain Lifescience GmbH, Nehren, Germany) assays. When these tests did not provide a species identification, sequencing of the 16S rRNA gene was performed as previously described [22]. Unfortunately, species and sub-species differentiation was not always achieved within the MAC and *M. chelonae-abscessus* complex groups.

**Table 1:** Clinical and microbiological criteria for diagnosis of NTM-PD as proposed in the 2007 ATS/IDSA statement

Clinical: - Pulmonary symptoms with nodular or cavitary opacities on chest radiograph or HRCT that show multifocal bronchiectasis with multiple small nodules. - AND sufficient exclusion of other diseases/ diagnoses.	
Microbiological: - Positive sputum culture results of at least two separately expectorated samples. - OR positive culture results from at least one bronchial wash or lavage. - OR transbronchial or other lung biopsy with mycobacterial histopathological features AND positive culture for NTM on biopsy or on at least one or more sputum samples or bronchial washing.	

Adapted from Griffith DE, Aksamit T, Brown-Elliott BA, Catanzaro A, Daley C, Gordin F, et al. An official ATS/IDSA statement: Diagnosis, treatment, and prevention of nontuberculous mycobacterial diseases. Am J Respir Crit Care Med. 2007;175(4):367–416

### Statistics

Continuous variables were described as medians and interquartile ranges (IQR) and categorical variables as absolute numbers and percentages of total. We compared cases with NTM-PD to cases not fulfilling the ATS/IDSA criteria using the Mann-Whitney U test for not-normally distributed continuous variables (age) and χ^2^ or Fisher’s exact test as appropriate (using odds ratios (OR) and 95% confidence intervals (CI 95%)) for dichotomous categorical variables. A two-sided error level of *p* < 0.05 was considered statistically significant. Multivariable logistic regression modelling was done including all factors associated with the outcome in univariate analysis. Starting from the full model including all co-variates, a backward stepwise selection process was performed retaining in the final model only variables with a *p*-value < 0.05. We checked for interactions with identified risk factors for those co-variates that seemed most plausible (gender and history of previous NTM) first and conducted a stratified Mantel-Haenszel analysis and tested for homogeneity of the ORs across the strata. For those factors with statistically significant (*p-*value < 0.05) heterogeneity (effect modification) an interaction term was included in the logistic regression model. However, none were retained (as statistically non-significant) in the final analysis. Statistical analysis was computed with SPSS version 25.0 (SPSS, Inc., Chicago, IL, USA) and Stata version 15 (StataCorp, Texas, USA).

## Results

**Fig. 1:**
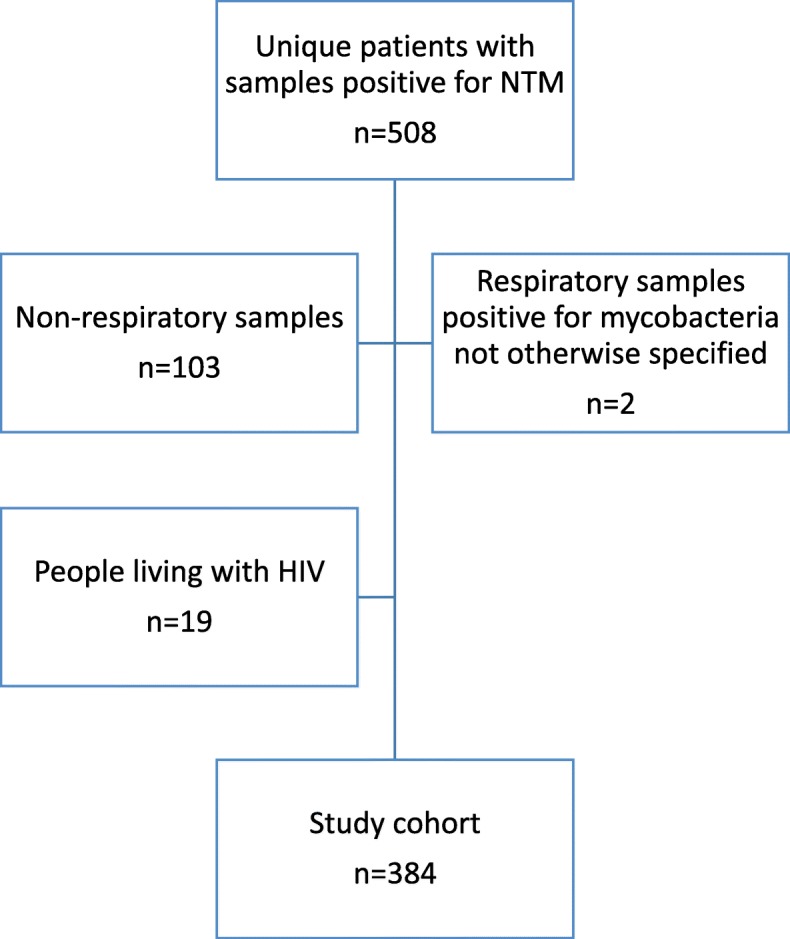
Selection of our study cohort

**Fig. 2:**
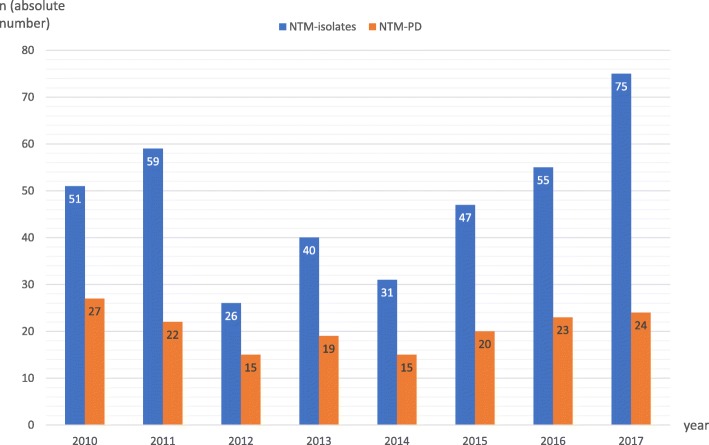
New isolates and diagnoses per year. Histogram with annual detection of new patients with an NTM-yielding respiratory sample (blue) and the annual incidence of new cases of NTM-PD (orange)

Of the 508 unique patients in whom NTM were isolated, we identified 384 patients with at least one NTM positive respiratory sample (Fig. 1). Figure 2 shows the evolution over time of NTM respiratory isolates and NTM-PD cases. We noted an increase in the number of respiratory NTM isolates from 51 in 2010 to 75 new isolations (in unique patients) in 2017, whereas the number of new cases of NTM-PD remained approximately stable during our study period. Over 90% of the patients in our cohort were seen by a pulmonologist. Forty-three percent (165/384) of the patients fulfilled ATS/IDSA criteria for NTM-PD. Table 2 presents baseline demographic, clinical, radiological and microbiological characteristics of the total cohort as well as by fulfilment of ATS/IDSA criteria. Over half of patients were current or ex-smokers (56.5%), 60.4% were male, and the majority were of Caucasian descent (95.8%), with a median (IQR) age of 65 (54–74) years. Pre-existing lung disease was present in 61%, most commonly COPD/emphysema (41.4%) and non-CF bronchiectasis (15.6%). Over 20% was immunocompromised to some extent. Besides previous episodes of NTM-PD and non-CF bronchiectasis, no other comorbidities were found to be significantly associated with NTM-PD after univariate analysis. These co-variates were not significant in multivariate analysis.

**Table 2:** Clinical characteristics of the 384 subjects of our study cohort by ATS/IDSA criteria

	Total cohort (*n* = 384)	ATS/IDSA + (*n* = 165)	ATS/IDSA - (*n* = 219)	OR^a^ [CI 95%]	aOR^b^ [CI 95%]
Demography	n (%)	n (%)	n (%)		
Median age in years (IQR)^c^	65 (54–74)	64 (52–74)	67 (55–75)	–	–
Male	232 (60.4%)	94 (57%)	138 (63%)	0.78 [0.51–1.17]	–
Smoking history^d^	217 (56.5%)	96 (58.2%)	121 (55.3%)	1.03 [0.68–1.57]	–
Comorbidity^e^					
Non-CF bronchiectasis	60 (15.6%)	33 (20%)	27 (12.3%)	**1.74 [1.00–3.03]**	**–**
Cystic fibrosis	24 (6.3%)	10 (6.1%)	14 (6.4%)	0.93 [0.40–2.14]	–
COPD/emphysema	159 (41.4%)	69 (41.8%)	90 (41.1%)	1.00 [0.66–1.51]	–
Previous NTM disease	31 (8.1%)	23 (13.9%)	8 (3.7%)	**4.19 [1.82–9.63**]	**–**
Previous TB disease	32 (8.3%)	12 (7.3%)	20 (9.1%)	0.76 [0.36–1.61]	–
Pulmonary aspergillosis	16 (4.2%)	7 (4.2%)	9 (4.1%)	1.01 [0.37–2.78]	–
Gastroesophageal reflux	28 (7.3%)	8 (4.8%)	20 (9.1%)	0.50 [0.21–1.16]	–
Active malignancy	63 (16.4%)	28 (17%)	35 (16%)	1.05 [0.61–1.81]	–
Immunodeficiency	91 (23,7%)	44 (26,7%)	47 (21.5%)	1.30 [0.81–2.09]	–
Transplantation	22 (5.7%)	9 (5.5%)	13 (5.9%)	0.90 [0.37–2.15]	–
Other	127 (33.1%)	52 (31.5%)	75 (34.2%)	0.86 [0.56–1.33]	–
Symptomatology^f^					
Fever	68 (17.7%)	40 (24.2%)	28 (12.8%)	**2.11 [1.24–3.61]**	**2.26 [1.12–4.56]**
Night sweats	30 (7.8%)	20 (12.1%)	10 (4.6%)	**2.80 [1.27–6.16]**	**–**
Fatigue/malaise	109 (28.4%)	63 (38.2%)	46 (21%)	**2.24 [1.43–3.53]**	**–**
Sputum	192 (50%)	90 (54.5%)	102 (46.6%)	1.31 [0.87–1.96]	–
Chest pain	41 (10.7%)	20 (12.1%)	21 (9.6%)	1.26 [0.66–2.41]	–
Cough	278 (72.4%)	135 (81.8%)	143 (65.3%)	**2.20 [1.35–3.59]**	**–**
Dyspoea	186 (48.4%)	77 (46.7%)	109 (49.8%)	0.84 [0.56–1.25]	–
Weight loss	108 (28.1%)	71 (43%)	37 (16.9%)	**3.59 [2.25–5.74]**	**2.47 [1.42–4.31]**
Hemoptysis	63 (16.4%)	35 (21.2%)	28 (12.8%)	**1.78 [1.03–3.07]**	**–**
Other	31 (8.1%)	11 (6.7%)	20 (9.1%)	0.69 [0.32–1.48]	–
Radiology (chest CT)^g^					
Fibrocavitary lesions	63 (16.4%)	46 (27.9%)	17 (7.8%)	**4.45 [2.44–8.12]**	**11.0 [4.74–25.57]**
Nodular bronchiectatic lesions	188 (49%)	103 (54.8%)	85 (38.8%)	**2.50 [1.64–3.81]**	**7.34 [3.87–13.91]**
Other	124 (32.3%)	15 (12.1%)	109 (49.8%)	**0.094 [0.052–0.17]**	**–**
Microbiology					
AFB staining positive	78 (20.3%)	64 (38.8%)	14 (6.4%)	**9.28 [4.96–17.34]**	**5.20 [2.60–10.42]**
*Pseudomonas aeruginosa*^*h*^	54 (14.1%)	24 (14.5%)	30 (13.7%)	1.07 [0.60–1.90]	–
*Aspergillus fumigatus*^*h*^	70 (18.2%)	42 (25.5%)	28 (12.8%)	**2.32 [1.37–3.93]**	**–**

Frequencies are given as absolute numbers (percentage of column total). ^a^Odds ratio and 95% confidence interval calculated from univariate analysis. Statistically significant differences in univariate analysis (two-sided *p* < 0.05) marked in bold. ^b^Adjusted odds ratio based on results from multivariate analysis. ^c^Age as median age (IQR) in years, no statistically significant difference with Mann-Whitney U test. Missing information for: ^d^12, ^f^6, ^g^9 patients respectively. ^h^Found as co-isolates with NTM

Cough (72.4%), sputum expectoration (50%) and dyspnoea (48.4%) were the most frequently reported presenting symptoms. Fever (17.7%), night sweats (7.8%), fatigue (28.4%), weight loss (28.1%), and hemoptysis (16.4%) were less common. In univariate analysis, fever (24.2%), night sweats (12.1%), fatigue (38.2%), cough (81.8%), weight loss (43%) and haemoptysis (21.2%) were more commonly associated with NTM-PD cases than with cases not fulfilling ATS/IDSA criteria (Table 2).

Fibrocavitary lesions were present in 63 (16.4%) patients, whereas 188 (49%) patients had nodular bronchiectatic changes; 16 (4.2%) had a normal chest CT. Fibrocavitary lesions were associated with NTM-PD (OR 4.45 [2.44–8.12]) as were nodular bronchiectatic lesions (OR 2.50 [1.64–3.81]).

Twenty percent of patients had positive AFB staining, which correlated significantly with ATS/IDSA criteria (OR 9.28 [4.96–17.34]). In univariate analysis (but not in multivariate analysis) co-isolation of *A. fumigatus* seemed significantly associated with NTM-PD cases (OR 2.32 [1.37–3.93]). This was not the case for co-isolation of *P. aeruginosa* (OR 1.07 [0.60–1.90]) (Table 2).

Multivariate analysis revealed that only symptoms like fever (OR 2.26 [1.12–4.56]) and weight loss (OR 2.47 [1.42–4.31]); fibrocavitary (OR 11.0 [4.74–25.57]) and nodular bronchiectatic findings (OR 7.34 [3.87–13.91]) on chest CT as well as positive AFB-staining (OR 5.20 [2.60–10.42]) were independently associated with NTM-PD.

**Fig. 3:**
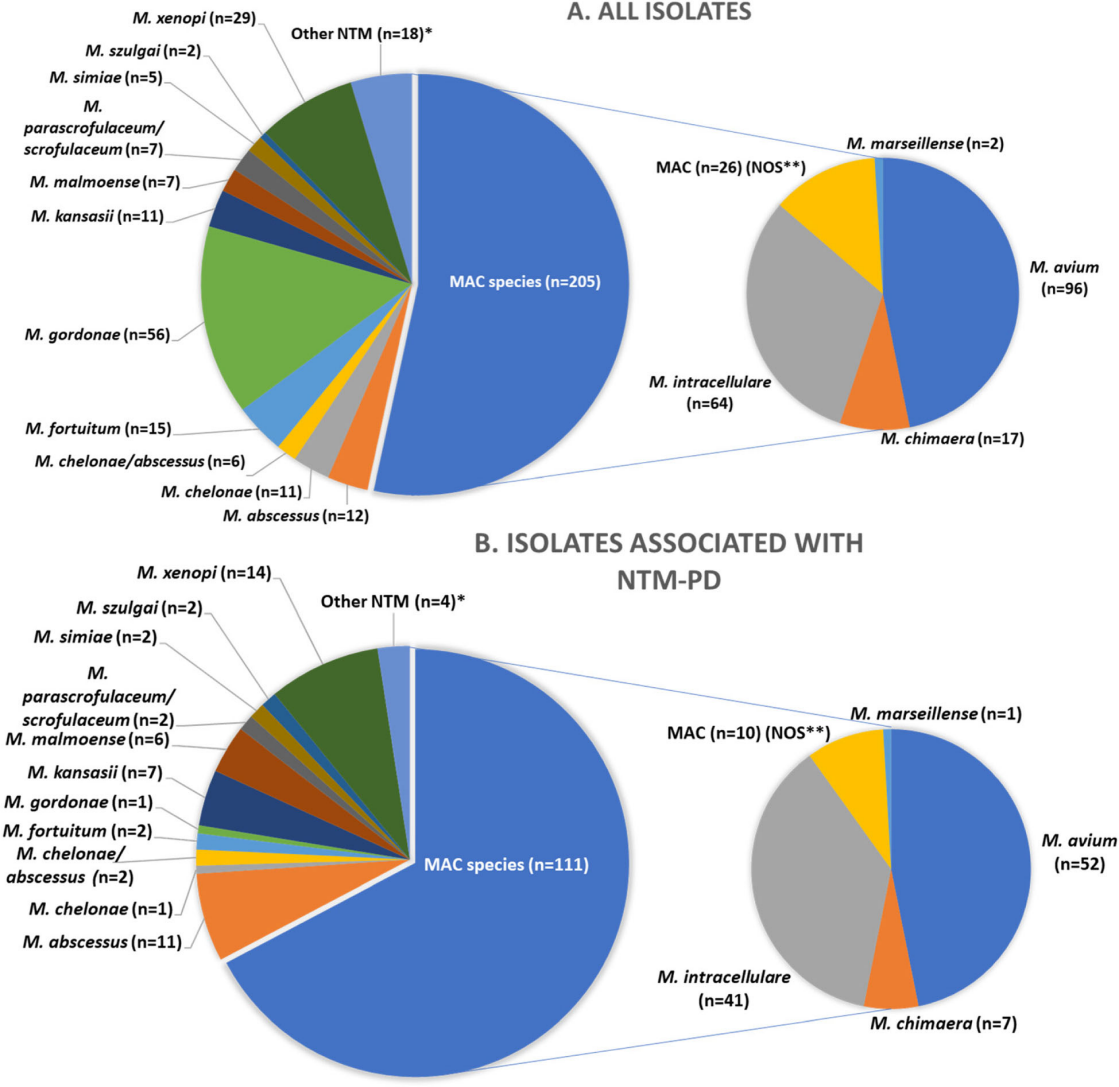
Frequencies of individual species in NTM isolates and diagnoses of NTM-PD. The upper pie chart shows all patients. The lower pie chart shows only patients meeting ATS/IDSA criteria for NTM-PD. *“Other NTM” encompasses all other, rarer isolates < 1% of total. Upper pie chart: *M. cellatum*; *M. florentinum*; *M. gilvum*; *M. holsaticum*; *M. lentiflavum*; *M. wolinsky/jacuzzi*; *M. noviomagense*; *M. novocastrense*; *M. peregrinum*; *M. terrae complex*. Lower pie chart: *M. cellatum*; *M. florentinum*; *M. noviomagense*; *M. peregrinum*. **NOS: MAC not further identified and reported as ‘MAC’

MAC was the commonest in terms of isolates in general and clinically relevant isolates (53.4%). On individual species level, *M. avium* (25%) was the most frequently isolated species followed by *M. intracellulare* (16.7%), *M. gordonae* (14.6%) and *M. xenopi* (7.6%). Among the clinically relevant isolates (NTM-PD), again MAC was predominant (67.3%); *M. avium* (31.5%), *M. intracellulare* (24.8%), *M. xenopi* (8.5%) and *M. abscessus* (6.7%) being the most frequently encountered pathogens in NTM-PD subjects (Fig. 3).

**Fig. 4:**
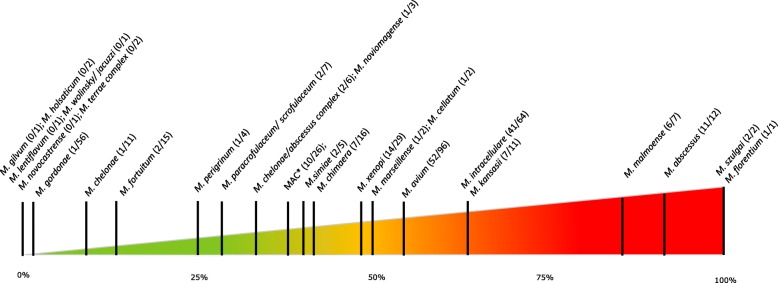
Ratio of clinical significance. Clinical significance per species; presented as a percentage based on the number of cases meeting ATS/IDSA criteria for NTM-PD and the total number of isolates per species. MAC and *M. chelonae/abscessus* complex, not further specified, were regarded separately. On the left, species of low virulence are found and on the right the most pathogenic species. Figure concept van Ingen et al., Thorax 2009, with permission

Figure 4 illustrates the pathogenic potential of the individual species according to ATS/IDSA criteria. *M. szulgai* (2/2) was rare but very pathogenic, followed by the more common *M. abscessus* (11/12), *M. malmoense* (6/7), *M. intracellulare* (41/64), *M. kansasii* (7/11), *M. avium* (52/96) and *M. xenopi* (14/29). *M. gordonae* (1/56), *M. chelonae* (1/9) and *M. fortuitum* (2/15) were rarely considered clinically relevant.

**Fig. 5:**
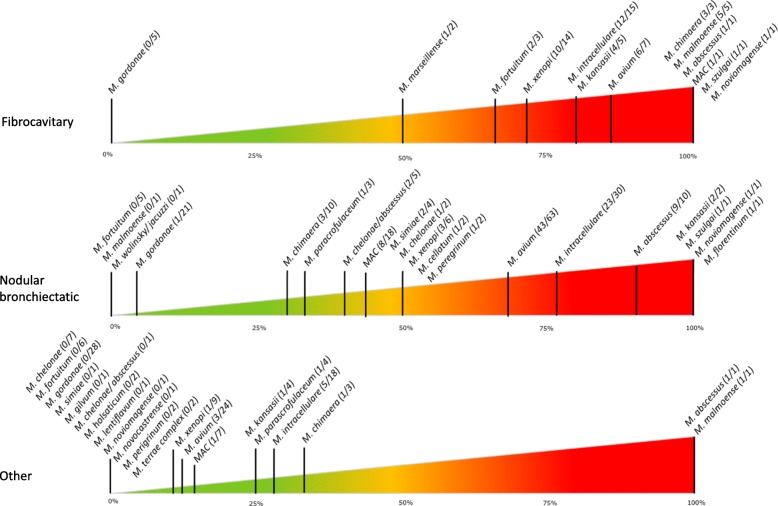
Ratio of clinical significance sorted by radiological presentation. Clinical significance per species; presented as a percentage based on the number of cases meeting ATS/IDSA criteria for NTM-PD and the total number of isolates per species. MAC and M. chelonae/abscessus complex, not further specified, were regarded separately. On the left, species of low virulence are found and on the right the most pathogenic species. In the upper bar cases with fibrocavitary changes are depicted, in the middle bar cases with nodular bronchiectatic changes are presented and in the lower bar cases with neither fibrocavitary nor nodular bronchiectatic changes are shown. Figure concept van Ingen et al., Thorax 2009, with permission

Most NTM-PD cases caused by *M. kansasii* (57.1%), *M. malmoense* (83.3%) and *M. xenopi* (71.4%) presented with fibrocavitary disease. In contrast, the majority of cases of *M. abscessus* related NTM-PD presented with nodular bronchiectatic disease (81.8%). This also applies to the majority of cases involving MAC; however, cases with MAC presenting with fibrocavitary lesions had a higher relative frequency of NTM-PD. When presenting with fibrocavitary changes on chest CT most isolated NTM were clinically significant (46/63; 73%)*.* As for nodular bronchiectatic lesions, this was less pronounced (103/188, 55%). When neither fibrocavitary nor nodular bronchiectatic lesions were present on chest CT, the isolated NTM was most often not considered clinically relevant (15/124; 12%) with few exceptions as presented in Fig. 5. As an example, *M. xenopi* was clinically relevant in 14 of 29 (48%) isolates. When associated with fibrocavitary lesions clinical relevance increased to 10/14 (71%), whereas in the presence of nodular bronchiectatic disease this was 3/6 (50%). When neither were present, *M. xenopi* isolation was mostly not relevant (only 1/9).

## Discussion

To the best of our knowledge, the present study is the first to describe clinical and radiographic features in relation to species-specific pathogenic potential of respiratory NTM isolates in Belgium. The frequency of NTM-PD remained stable over an 8-year time period in the three participating reference centres, despite increasing isolation frequency. NTM isolates were clinically significant in 43% of the patients. Symptoms like fever and weight loss, fibrocavitary and nodular bronchiectatic lesions on chest CT and a positive AFB stain were significantly associated with NTM-PD. MAC was the commonest mycobacterial isolate and pathogen in our cohort.

Although there was an annual increase in new NTM isolates, numbers of newly diagnosed NTM-PD remained stable. This evolution over time is in line with study findings in Denmark and the Netherlands [23, 24], whereas reports from the UK, Germany, Canada and the USA report an increasing incidence of NTM-PD [11, 13, 14, 25, 26]. The increasing isolation of NTM from respiratory samples may be partly due to a rising awareness and screening, but may also be explained by environmental factors or an ageing population with more comorbidities and predisposition to NTM-PD. However, the real incidence of NTM-PD remains uncertain since NTM-PD is not a reportable disease in most countries. Therefore, longitudinal and prospective registries could be very informative concerning the true incidence of NTM-PD.

Less than half of pulmonary NTM isolates are considered clinically relevant according to the ATS/IDSA criteria. Over 40% of patients in our cohort met the ATS/IDSA criteria, in contrast to lower reported numbers of 25–33% in similar studies from the Netherlands, the UK and South Korea [23, 27, 28]. This might be explained by the nature of referrals and underlying disease severity.

Patients in our study cohort were predominantly male and middle-aged, which is in concordance with some previous reports [6, 23] but in contrast to reports from the US and Japan where a different morphotype (white, middle-aged females) is described [12, 29, 30]. Pre-existing lung disease such as COPD and bronchiectasis were the most common comorbidities. Chronic respiratory disease is an important risk factor for NTM-PD, making it a pulmonologists issue: it has been associated with a 16-fold increased risk in a Danish population-based case-control study [31]. The finding was confirmed by Marras et al.*,* reporting a nine times higher incidence of NTM-PD in COPD [8]. Closer surveillance for NTM in patients with pulmonary disease may be warranted.

Diagnosis of NTM-PD according to ATS-IDSA criteria can be challenging as symptoms are often nonspecific and partly overlapping with underlying illness. Fever, night sweats and weight loss tend to be present in advanced NTM-PD [4]. Similarly in our study, fever and weight loss were independently associated with NTM-PD. Cough and fatigue also tended to be more frequently reported in patients with NTM-PD similar to findings from Aksamit et al., but these are rather non-specific symptoms which are often related to underlying diseases [4, 29]. The presence of nodular bronchiectatic lesions and especially fibrocavitary lesions on chest CT were significantly associated with NTM-PD. In the absence of either fibrocavitary or nodular bronchiectatic lesions, isolates were mostly not clinically relevant, a finding corroborating the ATS/IDSA criteria. Fibrocavitary lesions are a marker of severe NTM-PD with worse prognosis [4, 32, 33]. Similarly, the majority of patients in our cohort with respiratory samples positive on AFB staining suffered from NTM-PD. This is in line with previous studies, where AFB stain positivity has been associated with a higher bacterial burden, more severe NTM-PD and poor outcome [5, 33]. *A. fumigatus* co-isolation was also associated with NTM-PD in univariate analysis as suggested in previous studies by Kunst et al. and Provoost et al. [34, 35]. The value of this finding remains unclear as in multivariate analysis this factor was no longer deemed significant. Is *A. fumigatus* co-isolation a risk factor or the consequence of NTM-PD? It could also be related to underlying lung disease or immunosuppression [34–36]. Prospective studies or registries could identify true risk factors or indicators which could help refining the current ATS/IDSA criteria for establishing the diagnosis of NTM-PD.

With MAC accounting for more than half of the isolates, its prevalence in our study is comparable with a 2008 report from the Belgian national reference lab in which 38% of all isolates were MAC [18]. In other European studies, MAC was the predominant species as well, although regional differences were noted [15, 18]. *M. avium*, *M. intracellulare*, *M. gordonae* and *M. xenopi* were the most frequently isolated species, in accordance with a national report by Soetaert et al. where *M. avium*, *M. intracellulare* and *M. xenopi* accounted for 20, 21 and 15% of all (pulmonary and other) isolates respectively [17]. Of note, the same group previously showed that *M. chimaera* was frequently misidentified as *M. intracellulare* [22]. In our report we did not systematically re-evaluate these very closely related species. The difference for *M. xenopi* could be explained as our cohort did not properly cover the coast nor the southern half of the country which have a distinct environment. *M. xenopi* is a species mainly found in Europe with high concentrations in Southern Europe and along the English Channel [18, 37]. Accounting for only 3% of isolates, *M. abscessus* was rather rare which is similar to other European reports [12, 15]. Prevalence of *M. abscessus* isolates is substantially higher in Asia (16%) and Oceania (12%). These differences can be explained by different environmental factors and/or changing mycobacterial fauna over time [12, 15, 17, 18].

MAC was the main culprit (67.3%) for NTM-PD in this study cohort (Fig. 3). The position of MAC as the main cause of NTM-PD is established worldwide although frequencies vary largely between studies [12, 15]. Of individual species *M. szulgai*, *M. abscessus*, *M. malmoense*, *M. kansasii*, *M. intracellulare* and *M. avium* were clinically relevant in more than 50% of cases, suggesting a higher pathogenic potential than other species (Fig. 4). Compared to a Dutch study by Van Ingen et al.*,* there are several similarities but also some differences [23]. Our *M. abscessus* and *M. intracellulare* isolates were more frequently considered clinically relevant than their Dutch counterparts (91.6% versus 33 and 64% versus 12.5%). In our cohort *M. intracellulare* had a higher probability of clinical relevance than *M. avium* (64 and 54.2% respectively) in contrast to the Dutch report (12.5 and 40.7%) [23]. Our findings, however, are in line with other reports about a higher pathogenicity of *M. intracellulare* [15, 38]. These differences support the hypothesis of regional variation and illustrate the value of local data.

Clinical relevance varies not only by species but also by radiological presentation. Clinically significant isolates of *M. kansasii*, *M. malmoense* and *M. xenopi* mostly presented with fibrocavitary changes while *M. abscessus* and MAC related NTM-PD more often presented with nodular bronchiectatic changes, which has already been described [39–41]. Our findings corroborate previous suggestions that when assessing NTM clinical relevance, integration of radiological findings and species identification is useful [39, 40].

Our study has several limitations inherent to the retrospective study design. Selection bias cannot be excluded since data were collected from three major reference centres receiving the more complex cases. Additionally, increased awareness over time and more intense surveillance protocols for patients with pulmonary disease may have led to overestimation of NTM-PD risk. Our data are limited in scope, hence caution is warranted in generalising the findings. The lack of outcome data limit the predictive value of clinical significance.

## Conclusion

NTM are widely distributed environmental opportunistic pathogens which can cause significant morbidity and mortality especially in patient with underlying pulmonary conditions. In our cohort MAC species were the most frequently isolated, but *M. abscessus* and *M. malmoense* had a higher ratio of being clinically relevant when isolated. Because of geographical differences in isolates and clinical relevance between species, it is important to acknowledge local epidemiology as these data can be complimentary to the ATS/IDSA criteria in assisting clinicians in their treatment decision. Based on our study results, we suggest an added value for the AFB staining results, species identification and chest CT findings in the clinical decision-making process. Large multicentre prospective studies with standardized microbiological detection methods and clinical assessment reporting tools are highly needed.

## Data Availability

The dataset used and/analysed during the current study is available from the corresponding author on reasonable request.

## Abbreviations

AFB: Acid-fast bacilli
ATS: American Thoracic Society
CF: Cystic fibrosis
CI 95%: 95% confidence interval
COPD: Chronic obstructive pulmonary disease
HIV: Human immunodeficiency virus
IDSA: Infectious Diseases Society of America
IQR: Interquartile ranges
MAC: *Mycobacterium avium* complex
MTBc: *Mycobacterium tuberculosis* complex
NTM: Non-tuberculous mycobacteria
NTM-PD: Non-tuberculous mycobacterial pulmonary disease
OR: Odds ratio
